# The evolutionary dynamics of human influenza B virus in Bangladesh, 2007-2025

**DOI:** 10.1016/j.ijregi.2026.100999

**Published:** 2026-09-06

**Authors:** Suprova Islam, Mehedi Hasan

**Affiliations:** Department of Microbiology, Jagannath University, Dhaka 1100, Bangladesh

**Keywords:** Influenza B virus, Hemagglutinin, Neuraminidase, Evolutionary dynamics, Bangladesh

## Abstract

**Objectives:**

Influenza B virus causes significant seasonal respiratory illness, yet its long-term evolutionary data from South Asia remain limited. We investigated the evolutionary dynamics of influenza B viruses circulating in Bangladesh during 2007-2025.

**Methods:**

Complete hemagglutinin (HA) and neuraminidase (NA) gene sequences were analyzed using phylogenetic, evolutionary, demographic, selection pressure, amino acid variation, glycosylation, and genetic proximity analyses.

**Results:**

Phylogenetic analyses showed clear segregation into the B/Victoria (B/Vic) and B/Yamagata (B/Yam) lineages. B/Vic viruses diversified after 2019 into multiple V1A-derived subclades, whereas B/Yam remained genetically conserved before disappearing after 2019. Viruses maintained high similarity to season-matched vaccine strains. HA evolved faster than NA (2.48 × 10^−3^ vs 2.12 × 10^−3^ substitutions/site/year), with the highest rate observed in B/Vic HA. Viral effective population size expanded during 2014-2016 and 2017-2019, followed by a marked decline during 2019-2021. Purifying selection predominated, although episodic diversifying selection occurred mainly in B/Vic viruses. Amino acid diversification was concentrated within HA antigenic loop regions, while glycosylation profiles remained largely conserved.

**Conclusion:**

B/Vic viruses in Bangladesh exhibited substantially greater evolutionary flexibility than B/Yam, driven primarily by antigenic diversification of HA. Continued genomic surveillance is important for monitoring viral evolution and informing vaccine strain selection.

## Highlights

• Distinct evolutionary trajectories were observed in B/Victoria (B/Vic) and B/Yamagata.
• B/Vic diversified rapidly post-2019, whereas B/Yamagata remained conserved and disappeared thereafter.
• Hemagglutinin evolved faster than neuraminidase, particularly within the B/Vic lineage.
• Purifying selection predominated despite ongoing antigenic drift.

## Introduction

Influenza viruses continue to cause substantial global respiratory illness and death through annual epidemics and occasional pandemics, resulting in an estimated 3-5 million cases of severe disease and 290,000-650,000 respiratory deaths worldwide each year [1]. Among seasonal influenza viruses, influenza B virus is a major human respiratory pathogen, accounting for approximately 23% of laboratory-confirmed influenza infections globally and contributing substantially to the overall burden of influenza-associated disease [2]. The impact of influenza B is particularly pronounced among children and adolescents, although severe outcomes, including hospitalization and mortality, also occur in older adults [[2], [3], [4]]. Although influenza B lacks the extensive animal reservoir and pandemic potential characteristic of influenza A viruses, it remains an important cause of seasonal influenza morbidity and mortality and frequently co-circulates with influenza A viruses during annual epidemics [2].

Influenza B virus, a member of the *Orthomyxoviridae* family, contains a segmented negative-sense RNA genome that encodes the surface glycoproteins hemagglutinin (HA) and neuraminidase (NA). These proteins are central to viral attachment, release, and immune recognition [5]. Since the 1980s, influenza B viruses have diverged into two antigenically and genetically distinct lineages, namely the B/Victoria (B/Vic)/2/87-like and B/Yamagata (B/Yam)/16/88-like lineages [6]. The continued co-circulation of these lineages has complicated seasonal vaccine design because immunity to one lineage offers only limited protection against the other. This challenge ultimately led to the introduction of quadrivalent influenza vaccines containing strains from both lineages [7].

Influenza B viruses generally evolve more slowly than influenza A viruses, yet they continue to undergo substantial antigenic drift through the accumulation of mutations, reassortment events, and alterations in glycosylation patterns, particularly within antigenic regions of the HA protein [8,9]. Their evolutionary dynamics are shaped by several interacting factors, including host immune pressure, viral fitness, population immunity, and transmission bottlenecks. Previous studies have reported lineage-specific differences in evolutionary rate, genetic diversity, and antigenic drift, with the B/Vic lineage often showing greater genetic diversification than the B/Yam lineage [10,11]. More recently, the coronavirus disease 2019 (COVID-19) pandemic dramatically disrupted the global circulation of influenza viruses and coincided with a striking decline in the detection of B/Yam lineage viruses worldwide, raising concerns about the lineage’s apparent extinction [12,13].

Ongoing genomic surveillance remains essential for tracking influenza virus evolution, identifying emerging variants, monitoring antigenic drift, and informing vaccine strain selection. While the global evolution of influenza B viruses has been examined extensively, information from South Asia, including Bangladesh, remains comparatively limited despite the region’s high population density, persistent viral circulation, and substantial influenza burden. Bangladesh represents an important setting for influenza transmission because its tropical monsoon climate supports seasonal circulation, whereas dense population networks and extensive human mobility may facilitate continued viral spread and diversification [14,15]. Nevertheless, long-term molecular evolutionary investigations of influenza B viruses circulating in Bangladesh are still scarce.

In the present study, we investigated the evolutionary dynamics of influenza B viruses circulating in Bangladesh between 2007 and 2025 using phylogenetic, molecular evolutionary, selection pressure, amino acid variation, and glycosylation analyses of the HA and NA genes. We also assessed the genetic proximity between circulating viruses and World Health Organization (WHO)-recommended vaccine strains to examine lineage-specific evolutionary divergence and its potential implications for influenza control strategies.

## Methods

### Data set preparation

Complete HA and NA nucleotide sequences of influenza B viruses collected between January 2007 and December 2025 were retrieved from the Global Initiative on Sharing All Influenza Data (GISAID) EpiFlu database (https://gisaid.org/). Only complete sequences were retained for analysis, whereas sequences containing extensive ambiguous nucleotides (“N”) were excluded (Supplementary Table 1). The datasets were organized into three groups according to the objectives of the study. (1) For phylogenetic analysis, up to five sequences per year were randomly selected from each of the B/Victoria and B/Yamagata lineages when more than five sequences were available; otherwise, all available sequences from that year were included. In addition, all WHO-recommended seasonal vaccine strains from the study period, together with the reference strains B/Vic and B/Yam, were incorporated into the dataset. Altogether, 67 HA and 67 NA sequences were used for phylogenetic reconstruction (Supplementary Table 2). (2) For the evolutionary dynamics analyses, up to 15 sequences per year from each lineage were randomly selected when more than 15 sequences were available, while all available sequences were retained for years with fewer isolates. This dataset consisted of 212 HA and 212 NA sequences, including 121 B/Vic and 91 B/Yam strains (Supplementary Table 3). (3) Analyses of genetic proximity, selection pressure, amino acid variation, and N-linked glycosylation patterns were performed using the available Bangladeshi influenza B virus sequences. These analyses were conducted separately for the B/Vic and B/Yam lineages. HA and NA nucleotide sequences were used to estimate genetic proximity, whereas the corresponding protein sequences were examined for amino acid substitutions and variation in glycosylation sites. For the N-linked glycosylation analysis, sequences with ambiguous amino acid residues at the specific sites evaluated were excluded because the presence or absence of an N-linked glycosylation motif could not be reliably determined.

### Phylogenetic analyses

Rooted phylogenetic trees were reconstructed to examine the evolutionary relationships of influenza B viruses circulating in Bangladesh. Nucleotide sequences were aligned using MAFFT v7 (Kazutaka Katoh, Osaka University, Suita, Japan) [16]. Maximum-likelihood phylogenies were then inferred in MEGA v12.1.2 (Temple University, Philadelphia, PA, USA) [17] under the Hasegawa–Kishino–Yano model with invariant sites (HKY + I), identified as the best-fit nucleotide substitution model according to the Bayesian information criterion (BIC). Statistical support for the inferred tree topologies was evaluated using 1000 bootstrap replicates.

### Genetic proximity analysis

Genetic proximity analysis was carried out to evaluate sequence variability within the HA and NA genes of Bangladeshi influenza B viruses. Pairwise nucleotide distances were estimated in MEGA v12.1.2 [17] using the HKY + I model selected on the basis of the BIC. Divergence values were subsequently converted to percentage identity values using the formula (1 − *p*) × 100, where *p* denotes the estimated nucleotide distance. The mean of the highest and lowest percentage identity values was then calculated and reported.

### Evolutionary dynamics analyses

Evolutionary rates, expressed as nucleotide substitutions per site per year, together with the time to the most recent common ancestor (tMRCA), were estimated for the HA and NA gene segments of both the B/Vic and B/Yam lineages using the Bayesian Markov chain Monte Carlo (MCMC) framework implemented in BEAST v2.6.7 (University of Auckland, Auckland, New Zealand) [18]. The HKY + I substitution model was applied because more parameter-rich models failed to achieve satisfactory statistical convergence [8].

All datasets were analyzed under a Bayesian skyline coalescent framework with an uncorrelated lognormal relaxed molecular clock to account for the dynamic population structure and variable evolutionary rates characteristic of influenza viruses. MCMC chains were run for 100 million generations, with parameters and trees sampled every 10,000 steps. Convergence and parameter performance were assessed in Tracer v1.7.1 (University of Edinburgh, Edinburgh, UK) [19], where all estimated parameters achieved effective sample size values greater than 200.

Temporal signal within each dataset was examined by linear regression of root-to-tip genetic distances against sampling time using TempEst v1.5.3 (University of Edinburgh, Edinburgh, UK) [20]. Changes in effective population size through time were subsequently inferred using the Bayesian skyline reconstruction model implemented in Tracer v1.7.1 [19], generating non-parametric Bayesian skyline plots without assuming a predefined demographic history.

### Selection pressure analysis

Selection pressure acting on the HA and NA genes of Bangladeshi influenza B viruses was evaluated by estimating synonymous (*d*_S_) and non-synonymous (*d*_N_) substitution rates at individual codon sites using the Datamonkey server (Temple University, Philadelphia, PA, USA) (http://www.datamonkey.org/). Three complementary codon-based approaches were applied: Single-Likelihood Ancestor Counting (SLAC) [21], Mixed Effects Model of Evolution (MEME) [22], and Fast Unconstrained Bayesian AppRoximation (FUBAR) [23]. For SLAC and MEME analyses, sites with *P* ≤ 0.1 were considered significant, whereas FUBAR sites with posterior probabilities ≥ 0.9 were interpreted as being under selection pressure.

### Amino acid mutation analysis

Complete amino acid sequences of the HA and NA proteins were aligned against season-matched vaccine reference strains recommended for the corresponding influenza seasons using the ClustalW algorithm (EMBL-European Bioinformatics Institute, Hinxton, Cambridge, UK) implemented in BioEdit v7.7.1 (Ibis Biosciences, Carlsbad, CA, USA) [24]. Amino acid mismatches at individual positions were identified, and the frequency of each substitution was calculated on a yearly basis. Mutation frequencies were reported as the number of sequences carrying a given amino acid substitution during the corresponding influenza season.

### Prediction of N-linked glycosylation sites

Potential N-linked glycosylation sites within the HA and NA proteins were predicted using the NetNGlyc 1.0 Server (Technical University of Denmark, Kongens Lyngby, Denmark) (https://services.healthtech.dtu.dk/services/NetNGlyc-1.0/) [25]. Amino acid motifs containing the consensus sequon Asn-Xaa-Ser/Thr, where Xaa represents any amino acid except proline, were considered potential N-linked glycosylation sites. All predictions were generated using the server’s default parameters.

## Results

### Phylogenetic analyses

Maximum-likelihood phylogenetic analyses of the HA and NA genes showed clear segregation of Bangladeshi influenza B viruses into the B/Vic and B/Yam lineages, although differences in subclade structure were apparent between the two trees (Figure 1). In the HA phylogeny (Figure 1a), most B/Vic isolates clustered within the V1A clade. Viruses detected in 2019, however, diversified into the V1A, V1A.3, and V1A.3a subclades, indicating simultaneous circulation of multiple genetic groups. From 2020 onward, the majority of isolates grouped within V1A.3a.2 together with the vaccine strain (bold italicized in the tree) B/Austria/1359417/2021, consistent with continued diversification and replacement of earlier subclades. By contrast, B/Yam isolates formed a compact and strongly supported Y3 cluster with comparatively little internal divergence. Vaccine strains B/Phuket/3073/2013 and B/Wisconsin/1/2010 also clustered within this group, reflecting the relative genetic stability of the lineage over time. The NA phylogeny (Figure 1b) broadly mirrored the HA topology, particularly within the B/Yam lineage, where all Bangladeshi isolates again clustered in Y3. Greater complexity was observed within the B/Vic lineage, where a distinct group of viruses from 2016-2018 formed a separate cluster within V1A. Their discordant placement between the HA and NA trees may indicate a reassortment event involving acquisition of NA segments from a Y3-like background into B/Vic viruses.

**Figure 1:**
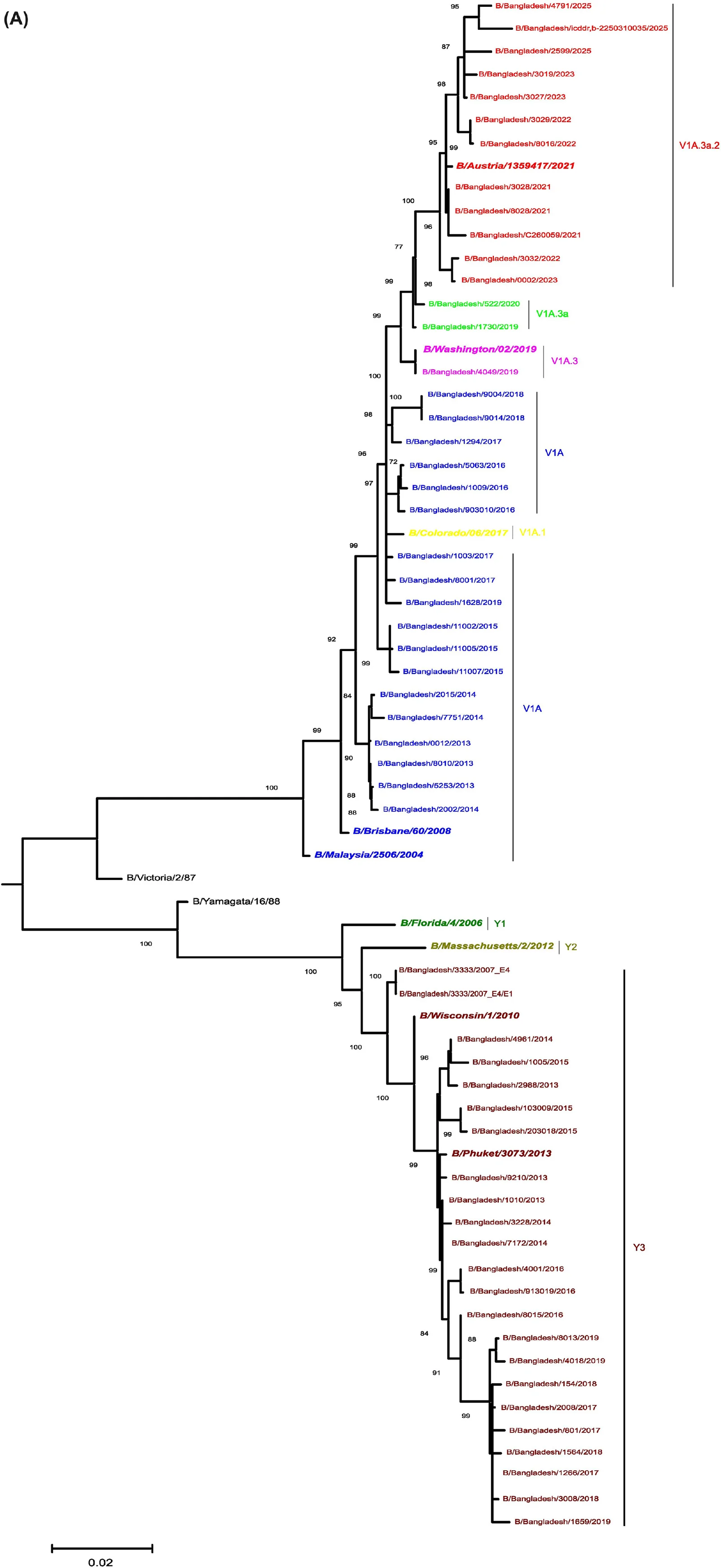

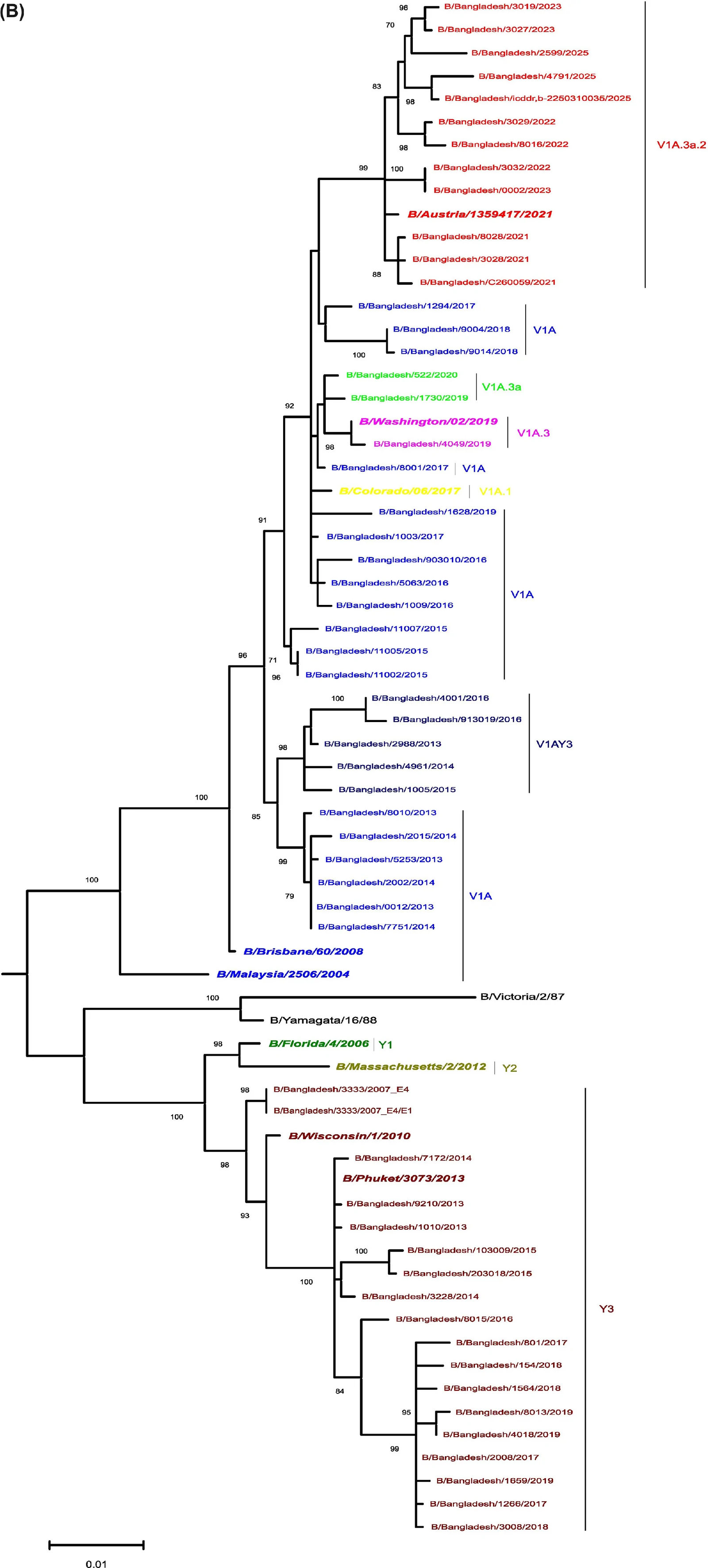
Maximum-likelihood phylogenetic trees of Bangladeshi influenza B viruses based on (a) HA and (b) NA nucleotide sequences. Trees were reconstructed using study isolates together with Northern Hemisphere vaccine strains from 2007 to 2025 as reference sequences (shown in bold italic). Tip labels indicate strain name and year of isolation, while clade designations are shown on the right. Bootstrap values ≥70% are displayed at key nodes. Scale bars represent nucleotide substitutions per site. HA, hemagglutinin; NA, neuraminidase.

### Genetic proximity

Genetic proximity between circulating Bangladeshi influenza B viruses and season-matched WHO-recommended vaccine strains was evaluated separately for the B/Vic and B/Yam lineages using both the HA and NA genes (Figure 2). Within the B/Vic lineage (Figure 2a), HA and NA sequences remained highly similar to vaccine strains throughout 2013-2025, with identity values generally ranging from 98.5% to 99.6%. A modest decline was observed during 2016-2017, followed by gradual recovery during 2018-2020. Another temporary reduction appeared in 2021, suggesting short-term divergence from matched vaccine strains, after which similarity increased again during 2022-2023 and remained high in 2025 despite minor fluctuations. No sequences were available for 2024. The B/Yam lineage (Figure 2b) showed greater year-to-year variability, particularly in the NA gene. HA similarity declined slightly between 2013 and 2015 before increasing sharply to 99.5% in 2016, whereas NA similarity remained comparatively lower until a pronounced increase in 2017. From that point onward, both genes stabilized at relatively high similarity levels (∼98.7-99.1%), although HA showed a slight decline from its 2016 peak.

**Figure 2:**
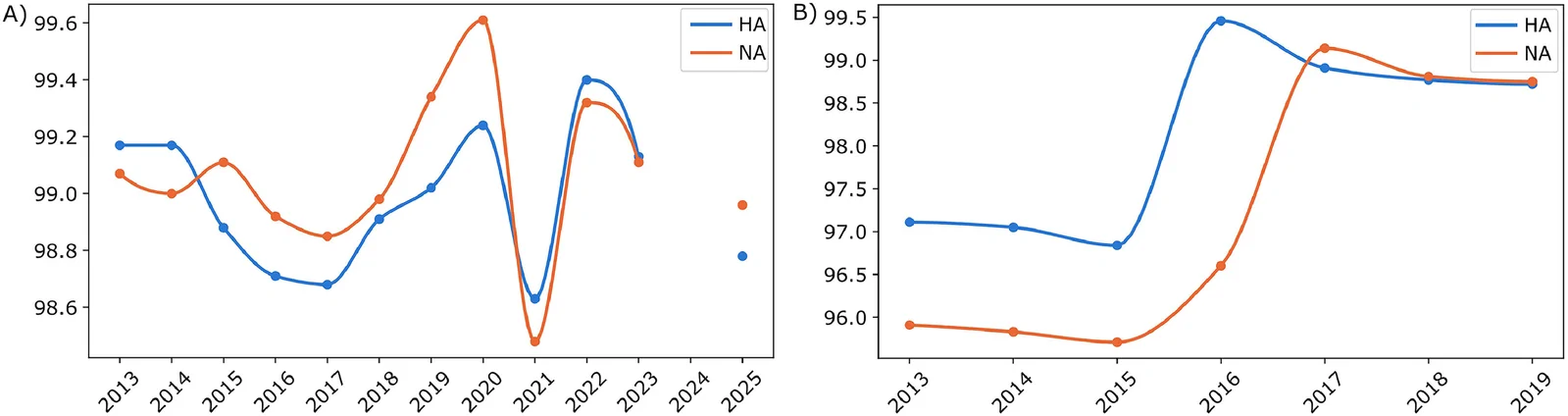
Genetic proximity of circulating Bangladeshi influenza B viruses to season-matched WHO-recommended vaccine strains for the (a) B/Vic lineage during 2013-2025 and (b) B/Yam lineage during 2013-2019. No B/Vic data were available for 2024. HA (blue) and NA (orange) are shown as annual data points with cubic-spline curves. The y-axes represent genetic proximity (%) to the corresponding vaccine reference strain, and the x-axes indicate the influenza season. B/Vic, B/Victoria; B/Yam, B/Yamagata; HA, hemagglutinin; NA, neuraminidase; WHO, World Health Organization.

### Temporal signal and evolutionary dynamics

Root-to-tip regression analysis performed in TempEst demonstrated a strong temporal signal in both the HA and NA datasets from Bangladesh, with perfect correlation between genetic divergence and sampling time (*R*^2^ = 1.0). These results supported the suitability of molecular clock–based evolutionary inference. Evolutionary analyses revealed distinct lineage-specific patterns in both genes (Table 1). The overall mean tMRCA was estimated to be October 2006 for HA and April 2007 for NA. Among the two lineages, B/Vic showed more recent origins for both HA (May 2012) and NA (November 2012), whereas B/Yam traced back to September 2006 for HA and January 2007 for NA. The HA gene evolved more rapidly overall, with a mean substitution rate of 2.48 × 10^−3^ substitutions/site/year (95% highest posterior density [HPD] interval: 2.11-2.87 × 10^−3^), compared with 2.12 × 10^−3^ substitutions/site/year (95% HPD interval: 1.84-2.41 × 10^−3^) for NA. Within the HA dataset, the B/Vic lineage showed the highest evolutionary rate (2.68 × 10^−3^ substitutions/site/year), whereas B/Yam evolved more slowly (1.99 × 10^−3^ substitutions/site/year). In contrast, the NA gene of the B/Yam lineage displayed a slightly higher substitution rate than that of B/Vic. Taken together, these findings point to relatively faster evolution of the HA gene, particularly within the B/Vic lineage circulating in Bangladesh.

**Table 1:** Divergence times and evolutionary rates of the HA and NA genes of influenza B viruses circulating in Bangladesh.

Gene	*n*	Mean tMRCA	Substitution rate (× 10^−3^/site/year)		
			Mean	Low 95% HPD	High 95% HPD
HA	212	Oct 2006	2.48	2.11	2.87
B/Vic HA	121	May 2012	2.68	2.25	3.13
B/Yam HA	91	Sep 2006	1.99	1.64	2.33
NA	212	Apr 2007	2.12	1.84	2.41
B/Vic NA	121	Nov 2012	2.07	1.72	2.42
B/Yam NA	91	Jan 2007	2.24	1.74	2.82

B/Vic, B/Victoria; B/Yam, B/Yamagata; HA, hemagglutinin; HPD, highest posterior density; *n*, number of sequences analyzed; NA, neuraminidase; tMRCA, time to the most recent common ancestor.

Bayesian Skyline reconstruction showed broadly similar demographic patterns for the HA and NA datasets over time (Figure 3). The inferred effective population size remained low and relatively stable from the late 2000s until around 2013, followed by a marked expansion beginning in 2014. Both genes showed major increases during 2014-2016 and again during 2017-2019, with the latter period representing the highest inferred effective population size across the study period, consistent with increased viral diversity and transmission. A sharp decline followed between 2019 and 2021, after which the effective population size stabilized at lower levels through 2025. The close agreement between the HA and NA Skyline patterns suggests synchronized evolutionary and population dynamics among circulating viruses.

**Figure 3:**
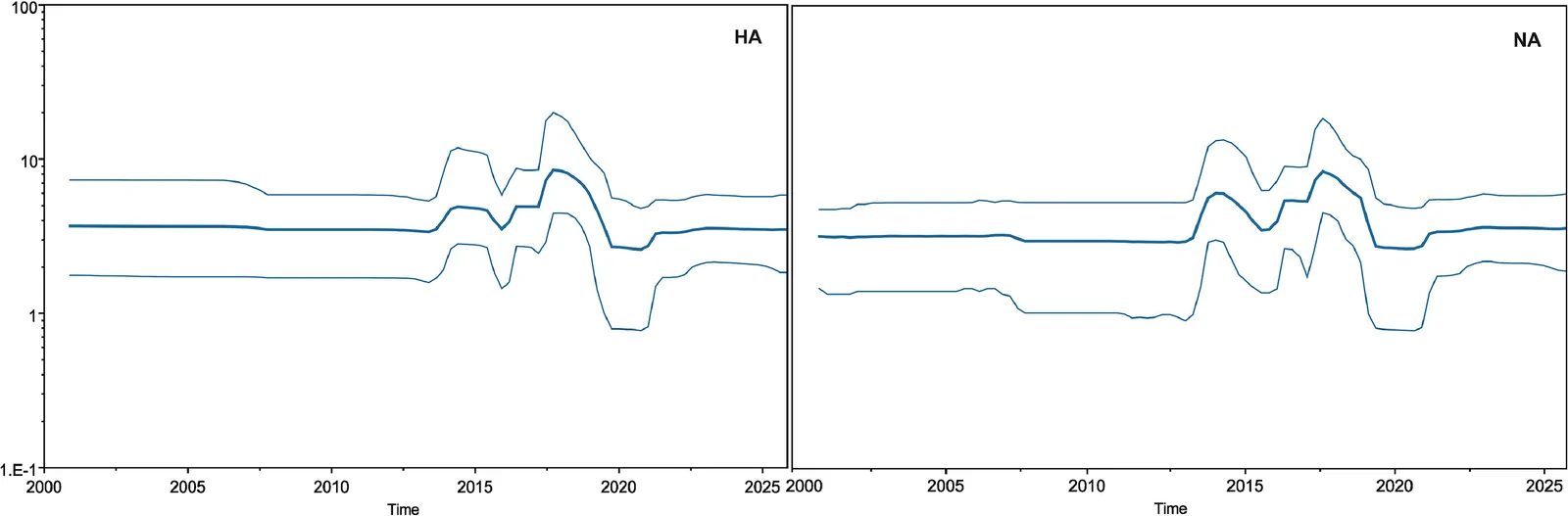
Bayesian Skyline reconstruction of temporal changes in viral effective population size inferred from HA and NA gene datasets of influenza B viruses circulating in Bangladesh during 2007-2025. The central solid line represents the mean effective population size, while the upper and lower bounds indicate the 95% HPD intervals. HA, hemagglutinin; HPD, highest posterior density; NA, neuraminidase.

### Selection pressure analysis

Selection pressure analyses of the HA and NA genes indicated an overall predominance of purifying to near-neutral selection, with mean d_N_/d_S_ ratios ranging from 0.57 to 0.97 across datasets (Table 2). The B/Vic lineage showed higher mean d_N_/d_S_ ratios than B/Yam for both HA (0.95 vs 0.57) and NA (0.97 vs 0.82). No positively selected codons were identified by SLAC in any dataset. MEME, however, detected numerous codons under episodic diversifying selection in both HA (109 sites) and NA (103 sites), with a greater number of positively selected sites in B/Vic than in B/Yam. FUBAR identified only a limited number of codons under pervasive diversifying selection. Detailed codon positions detected under positive selection are listed in Supplementary Table 4.

**Table 2:** Selection pressure analysis and positively selected codon sites identified in the HA and NA genes of influenza B viruses circulating in Bangladesh.

Gene	*n*	*d*_N_/*d*_S_	Number of sites under positive selection			Total sites
		SLAC	SLAC	MEME	FUBAR	
HA	812	0.90	0	109	3	585
B/Vic HA	605	0.95	0	71	1	585
B/Yam HA	207	0.57	0	20	2	584
NA	815	0.93	0	103	3	468
B/Vic NA	608	0.97	0	86	5	468
B/Yam NA	207	0.82	0	40	0	466

B/Vic, B/Victoria; B/Yam, B/Yamagata; *d*_N_, nonsynonymous substitution rate; *d*_S_, synonymous substitution rate; FUBAR, Fast Unconstrained Bayesian AppRoximation; HA, hemagglutinin; MEME, Mixed Effects Model of Evolution; *n*, number of sequences analyzed; NA, neuraminidase; SLAC, Single-Likelihood Ancestor Counting. SLAC and MEME analyses were performed using a significance threshold of *P* ≤ 0.1, while FUBAR analysis used a posterior probability threshold of ≥ 0.9.

### Amino acid variations

Amino acid variation in the HA and NA proteins was analyzed relative to lineage-specific vaccine reference strains across study years. HA mutations were categorized into the major antigenic loop regions (120-loop, 150-loop, 160-loop, and 190-helix) and non-loop regions (Figure 4; Supplementary Table 5), whereas NA mutations were examined across the full protein with particular attention to functionally important residues (Supplementary Table 6). Detected changes included substitutions, insertions (Ins), and deletions (Del). The B/Vic HA protein showed relatively limited variation during 2013-2018, with most changes consisting of low-frequency substitutions within the 120-loop. Between 2016 and 2017, I132V approached fixation, suggesting a selective sweep. Much greater diversification emerged after 2019, especially within the 150-loop region, where Ins177K/Ins178N and Del179 appeared alongside increasing frequencies of A142T, E143K, N144D, G144D/N, D144G/N, R148G, and N165K. Several of these mutations reached fixation by 2021. Additional substitutions detected during 2022-2025, including T136A/I, K177T, A181S, T194P/K, E195K, E196K, and V202I, further indicated ongoing antigenic drift. In contrast, non-loop regions showed only sporadic low-frequency substitutions without clear temporal expansion, suggesting that evolutionary pressure was concentrated mainly within antigenic sites. The B/Yam lineage remained considerably more conserved. Moderate- to high-frequency substitutions such as A123P, N131K, N180Y, and S165I were observed primarily during 2013-2015, after which variation became minimal. Both loop and non-loop regions remained largely stable over later years, with only occasional low-frequency substitutions and no detectable insertion or deletion events.

**Figure 4:**
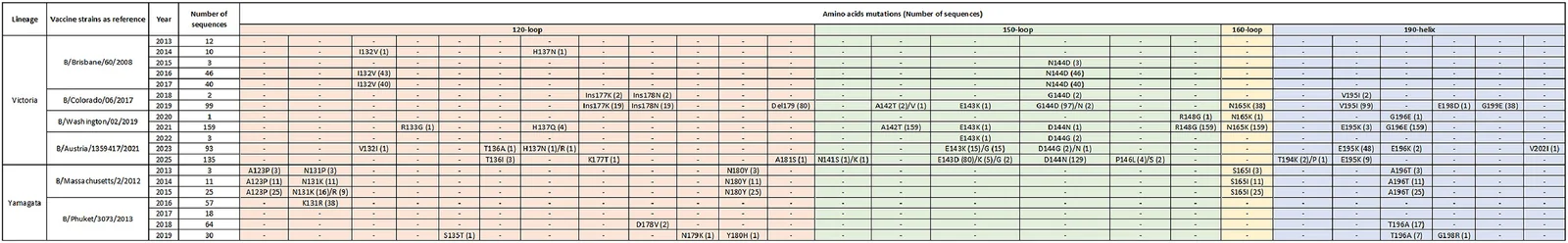
Temporal distribution of amino acid variations within antigenic loop regions of influenza B HA. Amino acid substitutions, insertions, and deletions are shown for B/Vic and B/Yam lineages relative to vaccine reference strains across the 120-loop, 150-loop, 160-loop, and 190-helix regions. Values in parentheses indicate the number of sequences carrying each mutation per year. Dashes (–) indicate no variation, and the total number of analyzed sequences is provided for each year. B/Vic, B/Victoria; B/Yam, B/Yamagata; Del, deletions; HA, hemagglutinin; Ins, insertions.

Compared with HA, the NA protein displayed lower overall variability. Substitutions were distributed more evenly across the protein and generally showed limited persistence over time. Catalytic and framework residues remained highly conserved across all years and lineages, consistent with strong functional constraints. Mutations associated with reduced neuraminidase inhibitor susceptibility were rare and appeared only sporadically at low frequencies. Most substitutions occurred within surface-exposed or stalk regions and appeared transient, indicating comparatively limited diversification of NA relative to HA.

### Prediction of N-linked glycosylation sites

N-linked glycosylation patterns in the HA and NA proteins of circulating influenza B viruses were analyzed across both lineages (Table 3a-d). The HA glycoprotein showed a largely conserved glycosylation profile in both B/Vic (*n* = 573) and B/Yam (*n* = 186) viruses. Within the B/Vic lineage (Table 3a), 11 potential glycosylation sites were identified. Positions 40 (NVTG), 74 (NCTD), 160 (NVTN), 248 (NQTE), 348 (NGTK), 533 (NITA), and 578 (NVSC) were present in at least 99% of sequences. Strong glycosylation predictions (+++/++) were consistently observed at positions 40, 74, 319 (NKSK), and 348, suggesting structurally conserved and potentially functional sites. The V1A.3a.2 clade (*n* = 370) retained a broadly similar glycosylation pattern without evidence of newly acquired sites. However, the 212 (NETQ) site, detected in 26% of B/Vic strains, was completely absent in V1A.3a.2 viruses, indicating clade-specific loss of glycosylation. In contrast, this same site was highly conserved in the B/Yam lineage (97.85%), highlighting lineage-specific divergence at an antigenically relevant region (Table 3b). Compared with HA, glycosylation patterns in NA were more conserved overall. In the B/Vic NA dataset (*n* = 571), six major glycosylation sites were identified (Table 3c), with positions 48 (NCTN), 94 (NLSE), 157 (NSSI), and 255 (NISN) detected in more than 98% of sequences. Among these, position 157 consistently showed strong prediction scores, suggesting an important structural or functional role in NA stability. By contrast, the N-terminal sites at positions 3 (NATF) and 7 (NYTN) displayed more variable prediction strengths, although strong predictions were more common within the V1A.3a.2 clade (∼51-53%), suggesting clade-associated modulation of N-terminal glycosylation potential. The B/Yam lineage (Table 3d) shared a broadly similar core NA glycosylation profile but also possessed additional variable sites at positions 379 (NGSN) and 405 (NGTW), which were absent in B/Vic strains, indicating lineage-specific diversification of NA glycosylation.

**Table 3:** Predicted N-linked glycosylation sites in the HA and NA proteins of influenza B viruses circulating in Bangladesh.

(a) B/Vic HA protein				
AA position(s)^a^	AA sequence(s)^b^	Prediction score	Total sequences *n* (%)	V1A.3a.2 *n* (%)
40-43	NVTG	+++/++	573 (100)	370 (100)
74-77	NCTD	+++/++	573 (100)	370 (100)
160-163	NVTN	+	573 (100)	370 (100)
181-184	NKTA	+	571 (99.65)	368 (99.46)
212-215	NETQ	+	149 (26)	0
248-251	NQTE	+	571 (99.65)	369 (99.72)
319-322	NKSK	+++/++	570 (99.48)	369 (99.72)
348-351	NGTK	+++/++	573 (100)	370 (100)
533-536	NITA	+	573 (100)	370 (100)
546-549	NHTI	+	567 (98.95)	368 (99.46)
578-581	NVSC	+	573 (100)	370 (100)
(b) B/Yam HA protein				
**AA position(s)**^a^	**AA sequence(s)**^b^	**Prediction score**	**Total sequences *n* (%)**	
40-43	NVTG	+++/++	186 (100)	
74-77	NCTD	+++/++	186 (100)	
160-163	NVTN	+	184 (98.92)	
181-184	NKTA	+	184 (98.92)	
212-215	NETQ	+++/++	182 (97.85)	
319-322	NKSK	+++/++	186 (100)	
348-351	NGTK	+++/++	186 (100)	
533-536	NITA	+	186 (100)	
546-549	NHTI	+	185 (99.46)	
578-581	NVSC	+	186 (100)	
(c) B/Vic NA protein				
**AA position(s)**^a^	**AA sequence(s)**^b^	**Prediction score**	**Total sequences *n* (%)**	**V1A.3a.2 *n* (%)**
3-6	NATF	+++/++	199 (34.85)	194 (52.57)
		+	371 (64.97)	175 (47.43)
7-10	NYTN	+++/++	197 (34.50)	190 (51.49)
		+	370 (64.80)	175 (47.43)
48-51	NCTN	+++/++	568 (99.47)	366 (99.19)
94-97	NLSE	+	566 (99.12)	369 (100)
157-160	NSSI	+++/++	570 (99.82)	368 (99.73)
255-258	NISN	+	562 (98.42)	367 (99.46)
(d) B/Yam NA protein				
**AA position(s)**^a^	**AA sequence(s)**^b^	**Prediction score**	**Total sequences *n* (%)**	
3-6	NATF	+	181 (97.31)	
7-10	NYTN	+++/++	124 (66.67)	
		+	62 (33.33)	
48-51	NCTN	+++/++	168 (90.32)	
94-97	NLSE	+	179 (96.24)	
157-160	NSSI	+++/++	179 (96.24)	
255-258	NISN	+	164 (88.17)	
379-382	NGSN	+	101 (54.30)	
405-408	NGTW	+	138 (74.19)	

AA, amino acid; B/Vic, B/Victoria; B/Yam, B/Yamagata; HA, hemagglutinin; *n*, number of sequences; NA, neuraminidase. Number of sequences analyzed—B/Vic HA (573; V1A.3a.2: 370), B/Yam HA (186), B/Vic NA (571; V1A.3a.2: 369), B/Yam NA (186); Prediction categories: +++/++ (high confidence), + (low).

a Amino acid position based on the reference strain B/Brisbane/60/2008 (accession number: EPI_ISL_246489).

b Asn-X-Ser/Thr motifs, where X represents any amino acid except proline.

## Discussion

Influenza B viruses constitute a significant cause of respiratory infections worldwide; however, their public health impact is often underestimated because of the dominant focus on influenza A viruses [3,[26], [27], [28]]. This study provides a long-term view of the evolutionary behavior, molecular evolution, and antigenic diversification of influenza B viruses circulating in Bangladesh over nearly 2 decades. The data showed sustained circulation and diversification of the B/Vic lineage, whereas the B/Yam lineage remained comparatively stable before disappearing from Bangladesh after 2019. Overall, the findings suggest that B/Vic possessed greater evolutionary flexibility than B/Yam, particularly within the HA gene, which appeared to drive much of the adaptive diversification observed during the study period.

The epidemiological patterns observed in Bangladesh are broadly consistent with observations from other regions, although the relative contribution of the two influenza B lineages has varied geographically and temporally. A global analysis of more than 1.8 million influenza cases from 31 countries during 2000-2018 showed that influenza B accounted for a median of 23.4% of influenza cases, with B/Vic tending to be more frequent in tropical countries and B/Yam more common in temperate regions [29]. In northern India, both lineages co-circulated during 2010-2016, with B/Yam predominating overall, whereas surveillance in Italy during 2004-2017 demonstrated alternating predominance and persistent co-circulation of the two lineages [28,30]. These observations highlight the marked geographic and temporal heterogeneity of influenza B epidemiology and provide context for the sustained B/Vic predominance and post-2019 disappearance of B/Yam, as observed in Bangladesh.

Phylogenetic analyses revealed lineage-specific clustering patterns that were largely consistent with global evolutionary trends reported for influenza B viruses. Most Bangladeshi B/Vic viruses clustered within the V1A clade before diversifying into V1A.3- and V1A.3a-related subclades after 2019, consistent with the emergence and subsequent predominance of V1A.3a.2 observed in global surveillance [31]. In contrast, B/Yam viruses remained tightly clustered within the Y3 clade, with relatively limited diversification before their disappearance after 2019. Differences between the HA and NA phylogenies among several B/Vic isolates collected during 2016-2018 may indicate reassortment events involving acquisition of NA segments from B/Yam-like ancestors. Similar reassortment events between influenza B lineages have been documented previously and may influence viral fitness and transmission dynamics [[32], [33], [34]].

Although both HA and NA retained high similarity to season-matched vaccine strains across most years, the B/Vic lineage showed clearer evidence of ongoing antigenic change. Short-lived declines in genetic proximity during 2016-2017 and again in 2021 likely corresponded to periods of accelerated drift preceding clade replacement. These shifts coincided with the relatively high evolutionary rate observed in the B/Vic HA gene and with the accumulation of substitutions within key antigenic loop regions. Several mutations that became increasingly common after 2019, including A142T, E143K, N144D, and Del179, have previously been linked to antigenic transitions among recently circulating B/Vic clades [28,35]. By comparison, B/Yam viruses remained more genetically conserved and accumulated fewer amino acid changes, consistent with their lower evolutionary rate and more restricted diversification.

The substitution rates estimated here fell within the range previously reported for influenza B viruses globally, typically between 1 × 10^−3^ and 3 × 10^−3^ substitutions/site/year [8,36]. HA evolved more rapidly than NA [37], likely because stronger immune pressure acts on the receptor-binding glycoprotein, whereas NA remains under tighter structural and functional constraint. Notably, the B/Vic lineage exhibited a higher evolutionary rate than B/Yam in the HA dataset, consistent with its greater genetic diversification, repeated clade turnover, and accumulation of antigenically important mutations [8,11,37,38]. In contrast, the lower evolutionary rate of the B/Yam lineage likely contributed to its comparatively conserved genetic structure throughout the study period. This contrast was also reflected in amino acid variation, which accumulated primarily within antigenically important regions of HA, whereas catalytic and framework residues in NA remained highly conserved. Mutations associated with reduced neuraminidase inhibitor susceptibility appeared only sporadically and at low frequency [39], suggesting that antiviral susceptibility has remained largely preserved among circulating strains.

The demographic reconstructions further illustrated the dynamic nature of influenza B circulation in Bangladesh. Bayesian Skyline analyses identified major increases in effective population size during 2014-2016 and 2017-2019, periods that likely reflected heightened viral transmission and diversification. The pronounced decline during 2019-2021 coincided with widespread COVID-19-related interventions, including travel restrictions, masking, and reduced social contact, patterns also observed in global influenza surveillance data [[40], [41], [42]]. Regional surveillance data from the WHO South-East Asia Region similarly demonstrated substantial changes in influenza circulation during the COVID-19 pandemic. Analysis of FluNet data showed that B/Vic was the predominant identified influenza B lineage from 2020 to 2023, whereas B/Yam circulation declined markedly from 2019 onward. However, the study also reported substantial gaps and inconsistencies in influenza surveillance and reporting during the pandemic period, particularly in epidemiological data submitted to FluID [43]. These findings provide regional context for the marked decline in effective population size observed in Bangladesh during 2019-2021, which coincided with the broader disruption of influenza circulation during the COVID-19 period.

Despite ongoing diversification, selection pressure analyses indicated that influenza B viruses were shaped mainly by purifying selection, reflecting strong functional constraints across viral proteins [38]. At the same time, episodic diversifying selection at multiple codon sites, particularly in the B/Vic lineage, points to intermittent adaptive changes concentrated within antigenically important regions of HA. The largely conserved glycosylation profiles of both HA and NA further emphasize the balance between immune-driven diversification and the structural constraints required to maintain viral fitness.

This study has some limitations. Sequence availability varied substantially across years, and no B/Vic sequences were available for 2024, limiting continuous assessment of evolutionary trends during that period. In addition, the study relied exclusively on publicly available genomic data, which may not fully capture the geographic and temporal diversity of influenza B viruses circulating in Bangladesh. Antigenic characterization assays, such as hemagglutination inhibition testing, were also not performed; consequently, antigenic interpretations were inferred indirectly from genetic variation. Finally, experimental validation of positively selected mutations and glycosylation changes was beyond the scope of the present study.

## Conclusion

Influenza B viruses circulating in Bangladesh displayed clear lineage-specific evolutionary patterns, characterized by rapid diversification of the B/Vic lineage and comparatively limited diversification of B/Yam before its disappearance after 2019. The HA gene emerged as the major target of adaptive evolution, particularly within antigenic loop regions associated with immune escape. Continued genomic surveillance integrating phylogenetic, antigenic, and evolutionary analyses will remain important for tracking emerging variants and supporting evidence-based vaccine strain selection strategies in Bangladesh and elsewhere.

## Funding

This research did not receive any specific grant from funding agencies in the public, commercial, or not-for-profit sectors.

## Ethical approval

This study analyzed publicly available secondary data with no identifiable individual-level information; therefore, ethical approval was not required. Study does not involve humans nor animal subjects.

## Declaration of generative AI and AI-assisted technologies in the writing process

ChatGPT (version 5.2) was used solely for language editing and refinement. All content was reviewed and validated by the authors, who take full responsibility for the accuracy, originality, and integrity of the manuscript.

## CRediT authorship contribution statement

**Suprova Islam:** Writing – review & editing, Writing – original draft, Methodology, Formal analysis, Data curation. **Mehedi Hasan:** Writing – review & editing, Writing – original draft, Visualization, Validation, Supervision, Formal analysis, Conceptualization.

## Declaration of competing interest

The authors have no competing interests to declare.

## Appendix Supplementary materials

**Supplementary Table S1**. Isolate information for influenza B virus strains from Bangladesh (2007–2025) included in the HA and NA gene analyses. **Supplementary Table S2.** Influenza B virus strains selected for HA and NA phylogenetic analyses. **Supplementary Table S3.** Influenza B virus strains selected for HA and NA evolutionary dynamics analyses. **Supplementary Table S4.** Positively selected codon sites identified in the HA and NA genes of influenza B viruses circulating in Bangladesh using SLAC, MEME, and FUBAR analyses. **Supplementary Table S5.** Amino acid variations in non-antigenic regions of influenza B HA relative to vaccine reference strains across study years. **Supplementary Table S6.** Comprehensive amino acid variations in the NA protein of influenza B viruses across study years. Image, application 1
